# Primary Melanoma of the Cervix Uteri: A Systematic Review and Meta-Analysis of the Reported Cases

**DOI:** 10.3390/biology12030398

**Published:** 2023-03-02

**Authors:** Konstantinos S. Kechagias, Marina Zafeiri, Konstantinos Katsikas Triantafyllidis, Georgios Kyrtsonis, Georgios Geropoulos, Deirdre Lyons, Laura Burney Ellis, Sarah Bowden, Apostolia Galani, Maria Paraskevaidi, Maria Kyrgiou

**Affiliations:** 1 Society of Meta-Research and Biomedical Innovation, London W12 0FD, UK; 2 Department of Metabolism, Digestion and Reproduction, Faculty of Medicine, Imperial College London, London SW7 2BX, UK; 3 King’s College Hospital NHS Foundation Trust, London SE5 9RS, UK; 4 Department of Nutrition and Dietetics, Royal Marsden Hospital, London SW3 6JJ, UK; 5 Department of General Surgery, Croydon University Hospital, Croydon, London CR7 7YE, UK; 6 Department of General Surgery, University College London Hospitals, London NW1 2BU, UK; 7 Imperial College Healthcare NHS Trust, London W2 1NY, UK

**Keywords:** melanoma, cervix uteri, cervical cancer, gynecological malignancy, melanosis

## Abstract

**Simple Summary:**

Melanoma is a malignant cancer mainly affecting the skin, although other unconventional sites have also been reported. In this study, we systematically reviewed the literature to identify cases of malignant melanoma affecting the cervix uteri. Our study included 96 reports, which comprised 137 patients. Our findings revealed that most patients presented with non-specific symptoms, including vaginal bleeding and discharge, and most were postmenopausal. The main therapeutic approach included surgical resection. Even though most patients were diagnosed at early stages, the prognosis was poor. Knowledge regarding the rare occurrence of malignant melanoma in the cervix and the increased awareness of clinicians can prevent misdiagnosis and ultimately improve the outcomes of patients developing this rare malignancy.

**Abstract:**

Primary malignant melanoma (MM) of the cervix uteri is a rare and aggressive malignancy of the female reproductive tract. Considering that clinical data on this cancer are scarce, we aimed to comprehensively examine the currently available literature and provide an overview of the reported cases of cervical MM focusing on the clinical characteristics, diagnosis and therapeutic management. We conducted a systematic review of the literature by screening three electronic databases until June 2022. The critical appraisal checklist provided by the Joanna Briggs Institute was employed to evaluate the overall quality of the studies. We included 96 reports, which comprised 137 patients diagnosed with MM of the cervix. The mean age of the patients was 56.5 (median: 58, age range: 33–88). Data regarding menopausal status were provided for 98 patients with 15 being premenopausal and 83 being postmenopausal. The most common presenting symptom was vaginal bleeding (83%, 100/121). Biopsy (either excisional or punch biopsy) was used as the first diagnostic modality in most of the patients (67%, 64/95), followed by cytology (18%, 17/95). In 74 cases, the FIGO staging system for cervical cancer was used with the most common stage being FIGO stage I (38%, 28/74), followed by FIGO stage II (36%, 27/74), FIGO stage III (19%, 14/74) and FIGO stage IV (7%, 5/74). Most of the patients were managed surgically (90%, 119/131) with a hysterectomy (either radical or total), and a salpingo-oophorectomy with/without lymphadenectomy was the most common approach utilized (40%, 48/119). The data of clinical outcomes were provided for 105 patients, of whom 61 died (58%, 61/105) and 44 survived (42%, 44/105). Knowledge regarding the rare occurrence of MM in the cervix and the increased awareness of clinicians can prevent clinical misdiagnosis and ultimately improve further the clinical outcomes of patients developing this rare malignancy.

## 1. Introduction

Malignant melanoma (MM) is a cancer derived from melanocytes [1]. Normal melanocytes are growth-limited, derived from the neural crest and are widely distributed in all cutaneous and most mucosal surfaces. Therefore, MM appears mainly in the skin and mucosa, with a preference for areas exposed to ultraviolet (UV) radiation [2]. Although MM represents a small percentage of skin cancers diagnosed each year (3%), it is accountable for an overwhelming proportion of the mortality related to skin malignancy (25%), indicating its aggressive nature [3]. More rarely, MM can emerge from extra-cutaneous anatomical sites, including the female reproductive tract, with five percent of MM in female patients derived from the vulva, ovary, uterus or cervix [4].

MM of the cervix is considered rare, accounting for less than 1% of cervical malignancies, with only sporadic cases reported in the literature since the 1880s [5]. Although primary MM of the cervix is localized to the cervix at the early stages, it infiltrates the uterosacral ligaments, pelvic wall, vagina and vulva and spreads to distant organs at advanced stages. Usually, affected patients have a poor prognosis due to a lack of early symptoms and specific signs [6].

Staging of the disease is mainly but not exclusively performed using the FIGO 2018 staging system which is also considered a prognostic factor for cervical MM [7]. Other prognostic factors, such as lymph vascular space involvement, tumor thickness, nodal status and neovascularization, have been also reported in the literature, but their value constitutes a matter of debate. Regarding therapeutic management, there are no established standards, and treatment is often based on the regimens used for cutaneous MM [8]. Radiotherapy and chemotherapy as adjuvant therapies in combination with various surgical treatments, such as radical hysterectomy and pelvic lymphadenectomy, have been previously proposed [9]. Alternative therapeutic options include the use of monoclonal antibodies targeting cytotoxic T lymphocyte-associated antigen 4 (CTLA4), programmed cell death 1 (PD-1) and the small molecule inhibitors of the MAPK signaling pathway (serine/threonine–protein kinase B-Raf [BRAF] and mitogen-activated protein kinase kinase) [10].

Considering that clinical data related to cervical MM are scarce, we aimed to comprehensively examine the currently available literature and provide an overview of the reported cases of MM in the cervix, focusing on the clinical characteristics, diagnosis and therapeutic management. We also performed an integrated analysis of the included cases with MM of the cervix uteri to identify factors affecting survival.

## 2. Methods

This review was reported based on the “Preferred Reporting Items for Systematic Reviews and Meta-Analyses” (PRISMA) guidelines [11].

### 2.1. Literature Search

Two reviewers (MZ and GK) independently searched PubMed, Web of Science and Scopus library databases from inception until June 2022. The search included the following terms: “Melanoma*” AND (“cerv*” OR “vagina*”). Alternative descriptions of the aforementioned terms based on MeSH terms were also used for the literature search. No restrictions regarding study design, geographic region or language were applied. A manual search of references cited in the selected articles and published reviews was also completed to elicit undetected studies. Google Scholar screening, customized Google searches and consultation with experts were also used to identify additional articles in the grey literature. Discrepancies in the literature search process were resolved by a third investigator (KSK).

### 2.2. Eligibility Criteria

We included studies that provided data for patients diagnosed with MM of the cervix uteri. All study designs were considered eligible for inclusion (e.g., case reports, case series, cohorts) if they provided individual patient data for cases diagnosed with the disease. Review articles, abstracts submitted in conferences and non-peer reviewed sources were not eligible for inclusion. Published dissertations were also excluded from the literature search to avoid inclusion of preliminary or incomplete data. Studies on in vitro and animal models were excluded.

### 2.3. Data Extraction and Handling

For all studies, patient data were retrieved and handled by two authors (MZ and GK), who independently conducted the data extraction. We collected the following information when available: sex, age, comorbidities, family history of cancer, presenting symptom and duration of symptoms, laboratory tests, primary diagnosis, imaging findings, staging, therapeutic management and clinical outcomes. Any disagreements were discussed and resolved by a third author (KSK).

### 2.4. Quality Assessment

Risk of bias (RoB) was independently assessed by two authors (KSK and KKT). The critical appraisal checklist provided by the Joanna Briggs Institute (JBI) was employed to evaluate the overall quality of case reports and case series. The assessment was based on the reporting of 8 different elements, namely, patient demographics, medical history, health status, physical examination and diagnosis, concomitant therapies, post-intervention health status and drug administration reaction interface. The studies were scored either based on “Yes”, “No” and “Unclear or Not/Applicable” depending on the availability of information for every element.

### 2.5. Data Synthesis

Given the descriptive nature of this review, we used descriptive statistics to report demographics and clinical characteristics, with means for continuous variables and rates/percentages for binary variables. We reported duration of symptoms as a range due to inconsistent and approximate reporting of this information across studies.

Kaplan–Meier survival curves were generated to estimate overall survival. The definition of overall survival was based on the definitions provided by the authors. The patients were divided in different subgroups based on menopausal status, therapeutic management, hysterectomy type and use of adjuvant treatment. Data with a *p* < 0.05 were considered statistically significant. Missing and unidentifiable data were excluded from the statistical analysis.

## 3. Results

### 3.1. Study Characteristics

**Figure 1:**
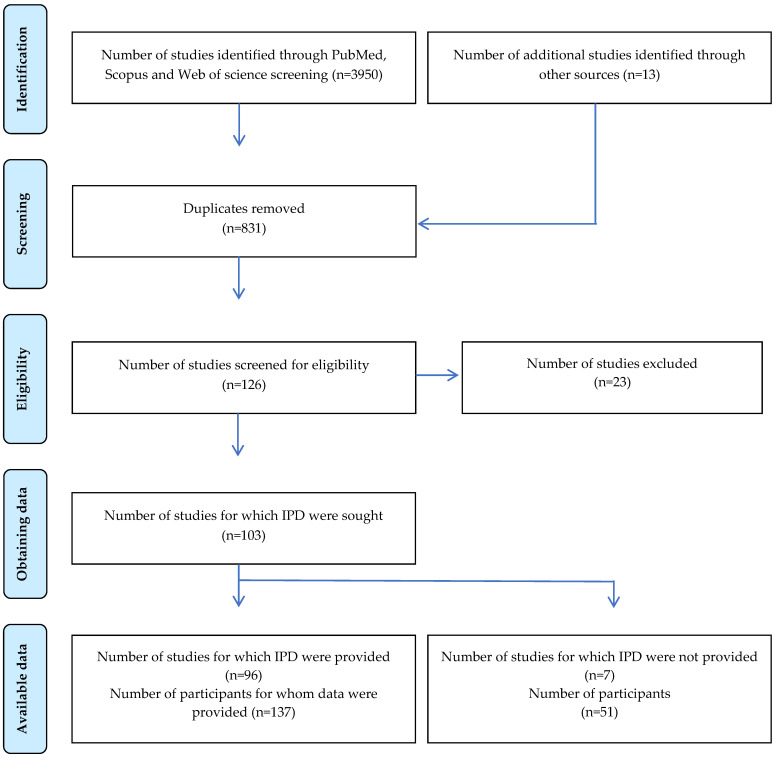
Prisma flowchart. IPD: individual patient data.

We identified 3950 articles through the literature search; a total of 831 bibliographic references were removed as duplicates, and 3006 articles were excluded as irrelevant through title and abstract screening. Two authors reviewed 113 full text articles for eligibility, and 13 articles were also retrieved from the reference lists of relevant narrative reviews. In the exclusion phase, seven studies (five retrospective cohorts and two prospective cohorts) that included 51 cases with primary MM of the cervix were excluded from the review because they did not provide individual patient data (Table S1). Finally, 96 studies [4,7,10,12,13,14,15,16,17,18,19,20,21,22,23,24,25,26,27,28,29,30,31,32,33,34,35,36,37,38,39,40,41,42,43,44,45,46,47,48,49,50,51,52,53,54,55,56,57,58,59,60,61,62,63,64,65,66,67,68,69,70,71,72,73,74,75,76,77,78,79,80,81,82,83,84,85,86,87,88,89,90,91,92,93,94,95,96,97,98,99,100,101,102,103,104] were found eligible for the systematic review (Figure 1). Overall, 47 of the studies were conducted in Asia, 27 in Europe, 19 in the Americas, 2 in Africa and 1 in Australia. In terms of design, 10 studies were case series, and 86 were case reports (Table S2). 

### 3.2. Patient Characteristics

**Figure 2:**
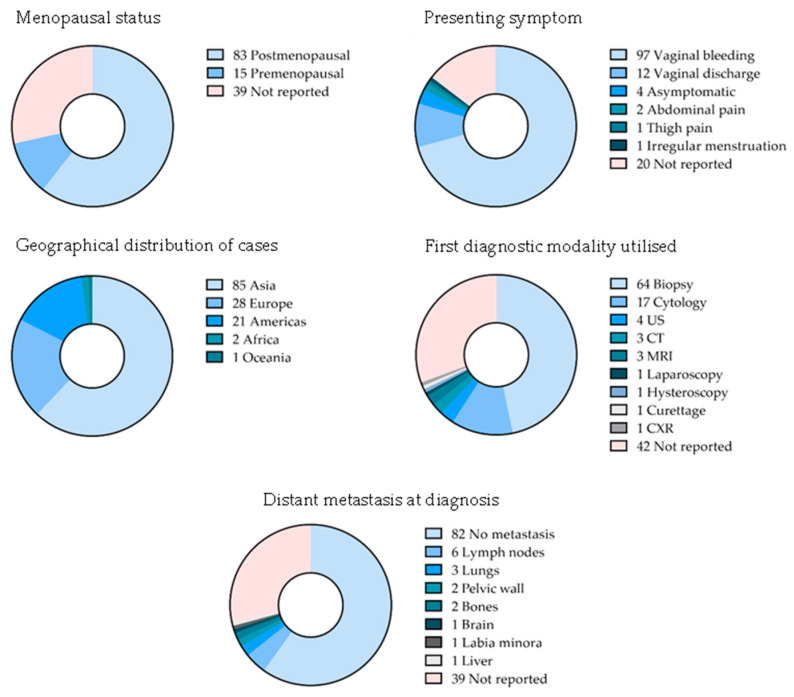
Characteristics of the included cases.

We identified a total of 137 cases of primary MM of the cervix uteri. The mean age of the patients was 56.5 years (median: 58, age range: 33–88). Data regarding menopausal status were provided for 98 patients, with 15 being premenopausal and 83 being postmenopausal. Data regarding presenting symptoms were available for 121 cases. The most common presenting symptom was vaginal bleeding (83%, 100/121) and its duration ranged from 4 days to 36 months. The second most common symptom was vaginal discharge (12%, 15/121), followed by abdominal pain (3%, 4/121) and thigh pain (1%, 2/121). A small fraction of patients remained asymptomatic prior to diagnosis (3%, 4/121) and were diagnosed secondary to abnormal cytology (Figure 2).

Data regarding diagnostics were provided for 95 patients. Biopsy (either excisional or punch biopsy) was used as the first diagnostic modality in most of the patients (67%, 64/95), followed by cytology (18%, 17/95), ultrasound (4%, 4/95), MRI (3%, 3/95) and CT (3%, 3/95). The diagnostic modalities used as secondary diagnostic or staging tools included CT (48%, 37/77), MRI (30%, 23/77) and PET-CT (19%, 15/77) (Figure 2).

Data on staging were provided for 86 patients. In 74 cases, the FIGO staging system of cervical cancer was used, with the most common being FIGO stage I (38%, 28/74), followed by FIGO stage II (36%, 27/74), FIGO stage III (19%, 14/74) and FIGO stage IV (7%, 5/74). Data regarding the presence of distant metastases at the time of diagnosis were provided for 98 patients. Metastatic sites included distant lymph nodes (6%, 6/98), lungs (3%, 3/98), bones (3%, 3/98), kidney (1%, 1/98), urinary bladder (1%, 1/98) and liver (1%, 1/98), while the majority of patients did not have a metastasis at the time of diagnosis (84%, 82/98).

Information on initial management was provided for 131 patients. Most of the patients were managed surgically (90%, 119/131) with a hysterectomy (radical or total), and a salpingo-oophorectomy +/− lymphadenectomy was the most common approach utilized (40%, 48/119). A small fraction of patients received neo-adjuvant radiotherapy (8%, 10/119) and chemotherapy (7%, 8/119). Chemotherapy +/− radiotherapy was used as the main therapeutic approach in a small number of patients (6%, 8/131) (Table S3).

### 3.3. Outcome and Survival Rates

**Figure 3:**
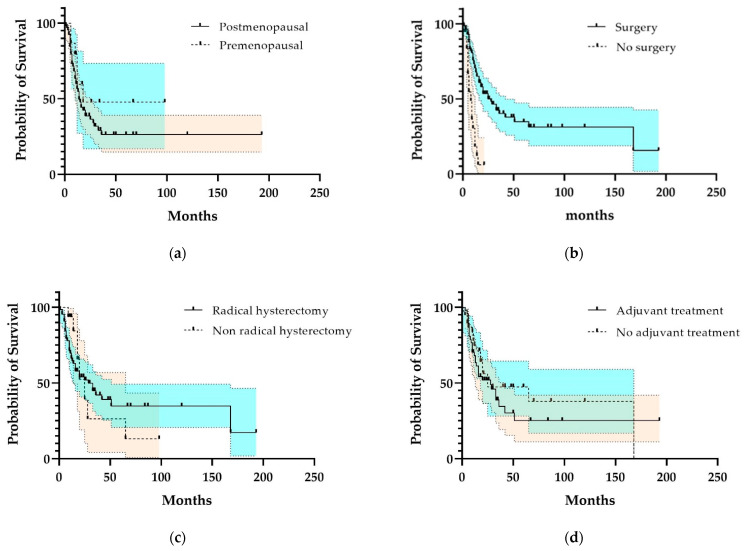
Kaplan–Meier survival curves for overall survival based on (**a**) menopausal status (postmenopausal vs. premenopausal), (**b**) management type (surgery vs. no surgery), (**c**) hysterectomy type (radical hysterectomy vs. non radical hysterectomy) and (**d**) supplementary treatment (adjuvant treatment vs. no adjuvant treatment). The prognosis was significantly improved following surgical management (including all types of surgical approaches) (*p* < 0.0001). The menopausal status, hysterectomy type and use of supplementary treatments were not associated with statistically significant differences in overall survival.

Data for clinical outcomes were provided for 105 patients, of whom 61 died (58%, 61/105) and 44 survived (42%, 44/105). The majority of deaths occurred within 12 months after the initial diagnosis (64%, 39/61). The follow-up period for survivors ranged from 4 to 193 months. Survival analysis indicated that the prognosis of patients was significantly improved following surgical management (including all types of surgical approaches) (*p* < 0.0001). The type of operation (radical hysterectomy vs. non-radical hysterectomy) did not show a statistically significant improvement in overall survival. Additionally, the overall survival was similar in patients with different menopausal statuses and in patients who received adjuvant chemotherapy or radiotherapy (Figure 3).

### 3.4. Quality of the Studies

Quality assessment of the eligible studies revealed that most of the recommended elements were fulfilled by the majority of included studies; thus, these were considered as low risk of bias. Nine studies attain a perfect score. The information most commonly marked as “Not/Applicable” across the studies was patients’ past medical history (Table S4).

## 4. Discussion

In this systematic review and meta-analysis of the literature, we identified cases of primary MM of the cervix uteri and examined their presentation, management and clinical outcomes. Our study included 96 reports, which comprised 137 patients diagnosed with MM of the cervix. Our findings revealed that most patients presented with non-specific symptoms, including vaginal bleeding and discharge, and most were postmenopausal. Therapeutic management included surgical resection with or without adjuvant chemotherapy in the majority of cases. Although most patients were diagnosed at FIGO stages I or II, the prognosis was poor with almost half of patients dying within the first 12 months after initial diagnosis and a large number experiencing recurrent disease.

### 4.1. Results in the Context of the Literature

The development of primary MM in the cervix was initially heavily questioned until the identification of melanocytes in the basal layer of the endocervix in 1959 [105]. The origin of the cells is not entirely clear, although it has been proposed that they originate from Schwann cells that have migrated from the neural crest [48]. These cells can give rise to rare pathological entities, such as cervical melanotic nevi and benign melanosis, a condition which is considered by many a precursor of MM of the cervix [46,106]. However, data on the subsequent risk of malignant melanoma following a diagnosis of benign melanosis are scarce, and further research is required to investigate the true association between these pathologic conditions and understand the potential for early detection at screening.

The presentation of the disease usually involves vaginal bleeding or discharge as cervical lesions can easily become ulcerated and/or infected [5]. However, cases of asymptomatic patients attending the clinic with abnormal cytology have been reported (Figure 2) [107]. Regarding diagnosis, the criteria described in the 1960s by Norris and Taylor are widely used by the majority of investigators. These criteria include (i) the presence of melanin in the normal cervical epithelium, (ii) the absence of other sources of melanoma in the body, (iii) the demonstration of junctional changes in the cervix and (iv) metastases according to the pattern of cervical carcinoma [108].

At the initial stages, the disease is limited to the cervical mucosa and then spreads locally to adjacent organs, such as the vaginal fornix, uterosacral ligaments, vulva and pelvic wall, following a similar pattern to other cervical malignancies [29]. Lymphatic metastasis also follows the typical pattern of drainage of other cervical carcinomas [27]. Although initially thickness was considered to be important as this is clinically relevant for MM of other sites [76], the International Federation of Gynecology and Obstetrics (FIGO) staging system for cervical cancer has now been more widely applied to MM of the cervix, as it correlates better with the pattern of spread and prognosis [8,44,76]. The FIGO staging system has also been characterized as the most reliable prognostic factor for primary MM of the cervix by Min et al. [9]. Cervical MM is considered to be human papillomavirus (HPV)-independent and distinct compared to other cervical epithelial malignancies [109,110,111,112], based on findings from matched controlled studies that examined the presence of different HPV subtypes in patients with malignant melanomas [113]. While the co-existence of cervical MM with other histological types of cervical cancer is pathophysiologically possible, it is rare based on the currently available literature. In this review, we identified only sporadic cases diagnosed with cervical MM and an additional synchronous epithelial cervical cancer, with the majority of patients suffering from primary cervical MM only.

Despite the aforementioned differences between MM and cervical epithelial malignancies, the management of cervical MM is mainly based on surgical approaches established for other histological types of cervical cancer [114]. Radical hysterectomy, salpingo-oophorectomy and pelvic lymphadenectomy are the cornerstones of therapeutic management with para-aortic lymphadenectomy being optional [24]. A recent integrated analysis of 149 cases confirmed the positive effect of hysterectomy-based treatments on survival [9]. Our review adds to the idea that radical hysterectomy is not superior to simple hysterectomy, although this result should be interpreted with caution due to the small number of patients included in the analysis (Figure 3). Many authors have also proposed a 2 cm clean surgical margin to optimize clinical outcomes, although this remains a matter of debate [38] along with the usefulness of pelvic lymphadenectomy [41]. Although data are limited on whether lymphadenectomy may or may not contribute to prolonged progression-free or overall survival, it has been advised in order to accurately stage disease and formulate recommendations for adjuvant treatment [77].

Chemotherapy with or without radiotherapy has been used sporadically as primary treatment in cases of unresectable tumors or patients with advanced disease [21]. Their effectiveness as adjuvant agents to surgery remains controversial, although many authors have used the aforementioned approach in patients with positive surgical margins or parametrial involvement [21]. Finally, immunotherapy and biological therapy have emerged the last decade as promising options for patients with primary MM of the cervix. Ipilimumab, a monoclonal antibody targeting cytotoxic T lymphocyte antigen-4 (CTLA-4), has been reported to improve overall survival with investigators commonly using it in combination with other agents, including antibodies targeting programmed death-ligand 1 (PD-1), BRAF, KIT, VEGF and MEK1/MEK2 mutations [115]. Other studies suggest that anti-PD-1 is associated with better overall survival compared to anti-CTLA4 in advanced or recurrent melanoma of the female reproductive tract [10]. Although these results have not been replicated in more recent reports, immunotherapy deserves further study not only for primary cervical MM but also for metastatic melanoma of the cervix [9]. While management guidelines are not officially established, a standardized treatment approach for patients with primary MM of the cervix was recently proposed by Wang et al.

The disease is associated with poor patient prognosis, especially if it is not diagnosed at an early stage [52]. Older studies have shown that the 5-year overall survival for patients is approximately 10%, and many patients die within three years of diagnosis (87.5%) [7]. This can be attributed, at least partially, to the inclusion of patients with disseminated disease. However, more recent reports revealed a higher 5-year overall survival of around 30% and a death rate of 65% within the first 3 years after initial diagnosis [9], possibly attributed to early diagnosis, aggressive surgical management and advancements in surgical techniques.

### 4.2. Strengths and Limitations

To the best of our knowledge, our study is the most comprehensive review of the reported cases of primary melanoma of the cervix uteri. Our findings present data from 137 patients and highlight published data with a quality assessment of the included studies.

However, our study is not without limitations. A broader drawback underlying our review is the low-quality nature of the case reports and case series included, which hinders the validity and scope of conclusions that can be reached. In fact, the potential risk of bias in these studies is inevitable, as these are especially vulnerable to the risks of overinterpretation and selection bias. In this way, their reported data, although intriguing, may be inaccurate and not representative of real-world statistics. Thus, data from larger cohorts and prospectively designed studies are needed to reach safer conclusions about the optimization of diagnostic and therapeutic management approaches used in patients with primary malignant melanoma of the cervix.

## 5. Conclusions

Malignant melanomas can arise at unconventional anatomical sites. In the cervix uteri, the tumor appearance can vary and thus may be confused with a primary epithelial malignancy. Although the associated prognosis is poor, aggressive surgical management in combination with alternative therapeutic options such as chemotherapy and immunotherapy may offer benefits to patients diagnosed with the disease. Knowledge of the rare occurrence of malignant melanoma in the cervix and the increased awareness of clinicians can prevent clinical misdiagnoses and ultimately improve the clinical outcomes of patients developing this rare malignancy.

## Data Availability

The data used to support the findings of this study are included within the article.

## Supplementary Materials

The following supporting information can be downloaded at: https://www.mdpi.com/article/10.3390/biology12030398/s1, Table S1: Cohort studies including patients with primary malignant melanoma of the cervix uteri without providing individual patient data; Table S2: Characteristics of the included studies; Table S3: Staging and therapeutic management of included patients; Table S4: Quality assessment of the included studies.
