# Supplementary material for: Primary Melanoma of the Cervix Uteri: A Systematic Review and Meta-Analysis of the Reported Cases
Source: Biology (Basel). 2023 Mar 2;12(3):398. doi: 10.3390/biology12030398 (PMC10045237; doi:10.3390/biology12030398)
Supplement: Supplementary file 1 [file biology-12-00398-s001.zip › biology-2151237-supplementary.pdf]

Table S1. Cohort studies including patients with primary malignant melanoma of the cervix uteri without providing individual patient data.

**Author**<br>**Year**	**Study design**	**Number of cases with primary malignant melanoma of the cervix**
Yano<br>2022	Retrospective cohort	24
Lewin<br>2021	Retrospective cohort	1
Tian<br>2021	Retrospective cohort	14
Pandey<br>2020	Prospective cohort	4
Udager<br>2017	Retrospective cohort	1
Karasawa<br>2014	Retrospective cohort	3
Tcheung<br>2012	Prospective cohort	4

Table S2. Characteristics of the included studies.

Author, Year, Country	Case	Age	Menopausal status	Presentation (duration of symptoms)	1st diagnostic modality	Other diagnostic modalities	Distant metastases at diagnosis	Recurrence after initial management	Outcome (status)
Asia									
Sone 2022 Japan	Case 1	73	post-menopausal*	Vaginal bleeding	Biopsy	- US<br>- MRI<br>- PET-CT<br>- CT	nil	- Brain, Lung, Liver, Bones<br>- Lymph nodes<br>(5 months post-surgery)	4 months disease free survival<br>7 months overall survival<br>(dead)
Ng 2021 China	Case 1	64	post-menopausal*	Asymptomatic	Cytology	- Radiological assessment (NOS)	nil	- Lung<br>(6 months post-surgery)	6 months disease free survival<br>11 months overall survival<br>(alive)
Suzuki 2021 Japan	Case 1	74	post-menopausal*	Vaginal bleeding	Cytology	- US<br>- MRI<br>- CT<br>- PET-CT	nil	- Lung, Pancreas, Liver, Peritoneum<br>(4 months post-surgery)	6 months overall survival<br>(dead)
Cai 2021 China	Case 1	48	N/A	N/A	N/A	N/A	N/A	N/A	N/A
	Case 2	42	N/A	N/A	N/A	N/A	N/A	N/A	N/A
	Case 3	55	N/A	N/A	N/A	N/A	N/A	N/A	N/A
	Case 4	42	N/A	N/A	N/A	N/A	N/A	N/A	N/A
	Case 5	34	pre-menopausal*	N/A	N/A	N/A	N/A	N/A	N/A
	Case 6	54	N/A	N/A	N/A	N/A	N/A	N/A	N/A
Anko 2020 Japan	Case 1	54	post-menopausal	Asymptomatic	Cytology	- Biopsy<br>- MRI<br>- PET-CT	nil	- Douglas pouch<br>(17 months post-surgery)	17 months disease free survival<br>50 months overall survival

									(alive)
Shakeel 2020 Pakistan	Case 1	68	post-menopausal	Vaginal bleeding (4 months)	N/A	N/A	nil	N/A	N/A
Pang 2019 China	Case 1	58	post-menopausal	Vaginal bleeding (10 days)	Biopsy	- CT	- Labia minora	nil	50 months disease free survival (alive)
Yin 2019 China	Case 1	55	post-menopausal	Vaginal bleeding (1 month)	Biopsy	N/A	nil	N/A	67 months disease free survival (alive)
	Case 2	81	post-menopausal*	Vaginal bleeding (6 months)	Biopsy	N/A	nil	N/A	21 months overall survival (alive)
Srivastava 2018 India	Case 1	42	N/A	Vaginal discharge and bleeding (12 months)	Biopsy	- CT<br>- PET-CT<br>- CXR<br>- 2nd biopsy	N/A	- Pelvic wall, Bones<br>- Lymph nodes (1 month post-surgery)	18 months overall survival (alive)
Sun 2018 China	Case 1	56	post-menopausal*	Vaginal bleeding (2 months)	N/A	N/A	nil	- Liver (29 months post-surgery)	36 month overall survival (dead)
	Case 2	62	post-menopausal*	Vaginal bleeding (1 month)	N/A	N/A	nil	- Recurrence (7 months post-surgery)	14 months overall survival (dead)
	Case 3	38	pre-menopausal*	Vaginal bleeding (2 months)	N/A	N/A	- Lymph nodes	N/A	Lost to follow up
	Case 4	62	post-	Vaginal	N/A	N/A	nil	N/A	70 months overall survival

		menopausal*	bleeding (1 month)					(alive)
Case 5	53	N/A	Vaginal bleeding (5 months)	N/A	N/A	nil	- Recurrence (NOS)<br>- Lymph nodes<br>(8 months post-surgery)	51 months overall survival (dead)
Case 6	57	post-menopausal*	Vaginal discharge (1 month)	N/A	N/A	nil	N/A	6 months overall survival (dead)
Case 7	80	post-menopausal*	Vaginal bleeding (12 months)	N/A	N/A	nil	N/A	3 months overall survival (dead)
Case 8	54	post-menopausal*	Vaginal bleeding (3 months)	N/A	N/A	nil	- Lung<br>(4 months post-surgery)	Lost to follow up
Case 9	50	N/A	Vaginal bleeding (5 months)	N/A	N/A	nil	N/A	Lost to follow up
Case 10	58	post-menopausal*	Vaginal bleeding (1 months)	N/A	N/A	nil	- Recurrence (NOS)<br>(3 months post-surgery)	20 months overall survival (dead)
Case 11	45	N/A	Vaginal bleeding (24 months)	N/A	N/A	nil	- Recurrence (NOS)<br>(2 months post-surgery)	3 months overall survival (dead)
Case 12	55	post-menopausal*	Vaginal bleeding (6 months)	N/A	N/A	nil	- Lung<br>(3 months post-surgery)	5 months overall survival (dead)
Case 13	60	post-	Vaginal	N/A	N/A	nil	N/A	5 months overall survival

			menopausal*	bleeding (1 month)					(dead)
	Case 14	69	post-menopausal*	Urinary incontinence (9 months)	N/A	N/A	nil	- Recurrence (NOS)<br>- Lymph nodes<br>(12 months post-surgery)	16 months overall survival (dead)
Kim 2018 Korea	Case 1	40	N/A	Vaginal discharge (7 months)	Punch biopsy	- CT<br>- MRI<br>- PET-CT	- Lung<br>- Bones	N/A	10 months overall survival (dead) *
Yuan 2017 China	Case 1	61	post-menopausal*	Vaginal bleeding	N/A	N/A	N/A	nil	193 months overall survival (alive)
	Case 2	74	post-menopausal*	Vaginal bleeding	N/A	N/A	N/A	- Skin<br>(24 months post-surgery)	33 months overall survival (dead)
	Case 3	56	post-menopausal*	Vaginal bleeding	N/A	N/A	N/A	nil	5 months overall survival (dead)
	Case 4	74	post-menopausal*	Vaginal bleeding	N/A	N/A	N/A	- Vagina<br>(12 months post-surgery)	28 months overall survival (dead)
	Case 5	77	post-menopausal*	Vaginal bleeding	N/A	N/A	N/A	- Vaginal recurrence<br>(12 months post-surgery)	25 months overall survival (dead)
	Case 6	45	N/A	Vaginal bleeding	N/A	N/A	N/A	nil	87 months overall survival (alive)
	Case 7	50	N/A	Vaginal	N/A	N/A	N/A	- Vagina	16 months overall survival

				bleeding				(2 months post-surgery)	(dead)
	Case 8	58	post-menopausal*	Vaginal bleeding	N/A	N/A	N/A	- Vagina (10 months post-surgery)	35 months overall survival (alive)
	Case 9	57	post-menopausal*	Vaginal bleeding	N/A	N/A	N/A	nil	4 months overall survival (dead)
	Case 10	42	N/A	Vaginal bleeding	N/A	N/A	N/A	- Skin (3.5 months post-surgery)	9 months overall survival (dead)
	Case 11	63	post-menopausal*	Vaginal bleeding	N/A	N/A	N/A	nil	10 months overall survival (dead)
	Case 12	54	N/A	Vaginal bleeding	N/A	N/A	N/A	- Skin (11.5 months post-surgery)	33 months overall survival (dead)
	Case 13	78	post-menopausal*	Vaginal bleeding	N/A	N/A	N/A	nil	12 months overall survival (dead)
	Case 14	68	post-menopausal*	Vaginal bleeding	N/A	N/A	N/A	- Skin (4.5 months post-surgery)	20 months overall survival (dead)
Noguchi 2017 Japan	Case 1	66	post-menopausal	Vaginal bleeding	Cytology	- MRI - PET-CT	nil	- Lung, Bones (6 months post-surgery)	13 months overall survival (dead)
Lim	Case 1	47	pre-	Irregular	Biopsy	- MRI	nil	N/A	N/A

2017 Singapore			menopausal	menstruation and vaginal discharge (recent onset)		- PET-CT			
Gupta 2016 India	Case 1	68	post-menopausal*	Vaginal bleeding	Biopsy	- MRI (brain)<br>- CT	nil	nil	60 months disease free survival<br>(alive)
Lee 2016 Korea	Case 1	70	post-menopausal*	Left thigh pain	MRI	- Biopsy	- Bones<br>- Urinary bladder	N/A	2 months overall survival<br>(dead)
Arık 2016 Turkey	Case 1	61	post-menopausal	Vaginal bleeding (4 months)	Cytology	- Punch biopsy	nil	- Lymph nodes<br>(12 months post-surgery)	12 months disease free survival<br>16 months overall survival<br>(dead)
Geredeli 2015 Turkey	Case 1	73	post-menopausal*	Vaginal bleeding	MRI	- PET-CT<br>- Biopsy	nil	- Heart<br>(4 monsths post initial management)	4 months disease free survival<br>7 months overall survival<br>(dead)
Cetinkaya 2015 Turkey	Case 1	43	N/A	Postcoital Vaginal bleeding	Biopsy	N/A	nil	nil	20 months disease free survival<br>20 month overall survival<br>(alive)
Mihmanli 2015 Turkey	Case 1	66	post-menopausal	Vaginal bleeding (2 months)	Biopsy	- CXR<br>- MRI	nil	N/A	N/A
Bhargava 2014	Case 1	35	N/A	Vaginal	Biopsy	N/A	N/A	N/A	N/A

India				bleeding and abdominal pain (2 months)					
Liu 2014 China	Case 1	65	post-menopausal*	Vaginal bleeding (1 week)	Incisional biopsy	- CXR - CT - US	nil	nil	30 months disease free survival 30 months overall survival (alive)
Min 2014 South Korea	Case 1	46	N/A	Postcoital vaginal bleeding (1 month)	Cytology	- Biopsy - MRI - PET-CT	nil	nil	24 months disease free survival 29 months overall survival (alive)
Omranipour 2014 Iran	Case 1	49	post-menopausal	Vaginal bleeding (2 months)	Biopsy	- CT	nil	N/A	N/A
Shrivastava 2014 India	Case 1	42	N/A	Vaginal bleeding (36 months) and right thigh pain	Biopsy	- Cystoscopy - Biopsy (bladder) - CXR - MRI	- Bones	N/A	5 months overall survival (dead)
Singh 2013 India	Case 1	35	pre-menopausal*	Vaginal bleeding and discharge (2 months)	Biopsy	- MRI - CXR	- Lymph nodes	N/A	6 months overall survival (dead)
Tsai 2012 Taipei	Case 1	66	post-menopausal	Vaginal bleeding (recent onset)	Biopsy	- MRI - PET-CT	- Lymph nodes	- Lymph nodes - Breast, Brain, Lung (2 months post-surgery)	2 months disease free survival
Zhang	Case 1	67	post-	Vaginal	Biopsy	- Cytology	nil	N/A	N/A

2011<br>China			menopausal	bleeding<br>(2 months)		- CXR<br>- CT			
Das<br>2010<br>India	Case 1	40	N/A	N/A	N/A	N/A	N/A	N/A	N/A
	Case 2	61	post-<br>menopausal*	N/A	N/A	N/A	N/A	N/A	N/A
Duggal<br>2010<br>India	Case 1	65	post-<br>menopausal	Vaginal<br>bleeding<br>(4 months)	Biopsy	N/A	nil	N/A	N/A
Khurana<br>2009<br>India	Case 1	58	post-<br>menopausal	Vaginal<br>bleeding<br>(1 month)	Punch<br>biopsy	- MRI	nil	- Lung-<br>Lymph nodes<br>(3 months post-surgery)	3 months overall survival<br>(alive)
An<br>2009<br>China	Case 1	67	post-<br>menopausal*	Vaginal<br>bleeding<br>(1 month)	Biopsy	- Radiological<br>assessment (NOS)	nil	- Lung<br>(6 months post-surgery)	N/A
Baruah<br>2009<br>India	Case 1	40	N/A	Vaginal<br>discharge<br>(5 months)	Biopsy	- CXR<br>- CT	nil	- Vagina<br>(18 months post- surgery)	18 months disease free survival<br>22 months overall survival<br>(alive)
Yücesoy<br>2009<br>Turkey	Case 1	61	post-<br>menopausal	Vaginal<br>bleeding<br>(4 months)	Biopsy	- Biopsy<br>- TAUS<br>- CXR<br>- IV pyelography	nil	nil	10 months disease free survival<br>10 months overall survival<br>(alive)
Mousavi<br>2006<br>Iran	Case 1	38	pre-<br>menopausal	Vaginal<br>bleeding<br>(3 months)	Biopsy	- CXR<br>- CT	nil	nil	24 months disease free survival<br>26 months overall survival<br>(alive)
Ma	Case 1	45	N/A	N/A	Biopsy	N/A	N/A	N/A	42 months disease free survival

2005<br>China		(mean)							(alive)
	Case 2		N/A	N/A	Biopsy	N/A	N/A	N/A	42 months overall survival<br>(dead)
	Case 3		N/A	N/A	Biopsy	N/A	N/A	N/A	6 months overall survival<br>(dead)
	Case 4		N/A	N/A	Biopsy	N/A	N/A	N/A	84 months disease free survival<br>(alive)
Gupta<br>2005<br>India	Case 1	39	pre-menopausal*	Vaginal bleeding and abdominal pain<br>(2 months)	Incisional biopsy	- US	nil	- Pelvis<br>- Lung, Liver, Urinary bladder<br>(4 months post diagnosis)	4 months disease free survival<br>6 months overall survival<br>(dead)
Kudrimoti<br>2004<br>India	Case 1	45	N/A	Vaginal discharge<br>(3 months)	Biopsy	N/A	N/A	N/A	N/A
Gupta<br>2003<br>India	Case 1	70	post-menopausal	Vaginal bleeding<br>(12 months)	Cytology	- Incisional biopsy	N/A	- widespread metastatic disease	4 months overall survival<br>(dead)
Deshpande<br>2001<br>India	Case 1	50	post-menopausal	Vaginal bleeding<br>(8 months)	Cytology	- Biopsy<br>- CT<br>- IV pyelography<br>- CXR	nil	nil	24 months disease free survival<br>24 months overall survival<br>(alive)
Furuya<br>2001<br>Japan	Case 1	33	pre-menopausal*	Vaginal discharge and back pain	Biopsy	N/A	nil	nil	6 months disease free survival<br>6 months overall survival<br>(alive)

Takehara<br>1999<br>Japan	Case 1	76	post-<br>menopausal	Vaginal<br>bleeding	Cytology	- Biopsy	nil	nil	30 months disease free survival<br>30 months overall survival<br>(alive)
Ishikura<br>1998<br>Japan	Case 1	51	N/A	Vaginal<br>bleeding	Biopsy	N/A	N/A	- Liver<br>- Peritoneum<br>(several months)	13 months overall survival<br>(dead)
Miyagi<br>1997<br>Japan	Case 1	57	post-<br>menopausal	Vaginal<br>bleeding<br>(recent onset)	nil	N/A	nil	- Peritoneum<br>(7m)	6 months disease free survival<br>18 months overall survival<br>(dead)
Moon<br>1993<br>Korea	Case 1	65	post-<br>menopausal	Vaginal<br>bleeding and<br>dysuria<br>(4 months)	CT	- MRI	N/A	N/A	N/A
Khoo<br>1991<br>Hong Kong	Case 1	62	post-<br>menopausal	Vaginal<br>bleeding<br>(6 months)	Biopsy	N/A	nil	- Lung, Brain<br>(13 months/14 months post-diagnosis)	14 months overall survival<br>(dead)
	Case 2	60	post-<br>menopausal	Vaginal<br>bleeding<br>(4 days)	Biopsy	N/A	- Pelvic wall	- Liver<br>(10 months post-diagnosis)	10 months overall survival<br>(dead)
	Case 3	37	pre-<br>menopausal*	Vaginal<br>bleeding	Biopsy^	N/A	nil	nil	10 months overall survival<br>(alive)
Chua<br>1989<br>Singapore	Case 1	52	N/A	Vaginal<br>bleeding<br>(2 months)	Excisional<br>biopsy	- CT	nil	- Labia, Kidney,<br>Mesentery<br>- Lymph nodes<br>(4 months post-surgery)	4 months disease free survival<br>25 months overall survival<br>(dead)

Europe									
Diakosavvas 2020 Greece	Case 1	34	pre-menopausal*	Asymptomatic	Incisional biopsy	- MRI<br>- PET-CT<br>- US	nil	- Lung<br>- Liver<br>(9 months post-surgery)	9 months disease free survival<br>12 months overall survival<br>(dead)
Pumpure 2020 Latvia	Case 1	25	pre-menopausal	Abdominal pain	Laparoscopy (+biopsy)	- CT<br>- US	- Omentum<br>-Lymph nodes	- Urinary bladder, Mesentery, Ovary<br>- Lymph nodes<br>(12 months post-surgery)	67 months overall survival<br>(alive)
Indini 2019 Italy	Case 1	61	post-menopausal*	N/A	N/A	N/A	nil	- Liver, Lung<br>(7 months post-surgery)	7 months overall survival<br>(dead)
Eniu 2019 Romania	Case 1	51	N/A	N/A	Biopsy	- Imaging (NOS)	nil	- Lungs, Bones<br>(12 months post-surgery)	12 months disease free survival<br>20 months overall survival<br>(dead)
Julião 2017 Portugal	Case 1	64	post-menopausal	Vaginal bleeding and dyspareunia	US	- Biopsy<br>- CT<br>- MRI	nil	- Lymph nodes<br>- Liver<br>(12 months post-surgery)	20 months overall survival<br>(alive)
Ferraioli 2016 France	Case 1	74	post-menopausal*	N/A	N/A	- CT	nil	- Lung, Brain	5 months disease free survival<br>11 months overall survival<br>(dead)
Shenjere 2014 UK	Case 1	51	N/A	Vaginal bleeding	CT	- MRI<br>- Biopsy<br>- CT	- Lymph nodes	- Pelvis<br>- Abdominal scar<br>(10 months post diagnosis)	10 months overall survival<br>(alive)

Myriokefalitaki 2013 UK	Case 1	63	post-menopausal	Vaginal discharge and bleeding (4 months)	Biopsy	- CT - MRI	nil	nil	40 months disease free survival 40 months overall survival (alive)
Parada 2012 Spain	Case 1	76	post-menopausal	Vaginal Bleeding (6 months)	Biopsy	- CXR - Cystoscopy - Colonoscopy	nil	N/A	N/A
Simões 2011 Portugal	Case 1	75	post-menopausal*	Vaginal bleeding (1 month)	Incisional biopsy	- CT - PET-CT	nil	- Liver - Bones (2 months post-surgery)	2 months disease free survival 5 months overall survival (dead)
Mandato 2009 Slovenia	Case 1	61	post-menopausal	Vaginal spotting (some weeks)	Biopsy	N/A	nil	nil	120 months overall survival (alive)
Pusceddu 2008 Italy	Case 1	43	N/A	Vaginal discharge Vaginal bleeding	MRI	- Biopsy	nil	- Vulva, Vagina (43 months post initial management)	43 months disease free survival 65 months overall survival (dead)
Wydra 2006 Poland	Case 1	54	N/A	Vaginal bleeding (4 months)	Biopsy	N/A	nil	- Vagina (3 months post-surgery)	8 months overall survival (dead)
Siozos 2005 UK	Case 1	82	post-menopausal*	Vaginal bleeding (6-8 weeks)	Punch biopsy	- MRI - CT	nil	N/A	N/A
Makovitzky	Case 1	67	post-menopausal	Vaginal bleeding	Biopsy	- Pap smear - CT	nil	nil	12 months disease free survival

2003<br>Germany						- PET-CT			12 months overall survival<br>(dead)
Boldt<br>2003<br>Germany	Case 1	26	pre-<br>menopausal	N/A	US	- Scintigram<br>- MRI<br>- CT	nil	N/A	11 months disease free survival<br>11 months overall survival<br>(alive)
	Case 2	70	post-<br>menopausal*	Abdominal pain	Cytology	- CT<br>- MRI	nil	N/A	5 months disease free survival<br>5 months overall survival<br>(alive)
Benson<br>2000<br>UK	Case 1	73	post-<br>menopausal	Vaginal discharge and bleeding<br>(2 months)	Biopsy	N/A	nil	- Urethra<br>(8 weeks post diagnosis)	8 months overall survival<br>(dead)
Wasef<br>1999<br>UK	Case 1	71	post-<br>menopausal	Vaginal<br>bleeding	Hysteroscopy	- Biopsy<br>- Radiological assessment (NOS)	nil	- Ascites<br>(5 weeks post-surgery)	3 months overall survival<br>(dead)
Butt<br>1996<br>UK	Case 1	72	post-<br>menopausal	Vaginal<br>bleeding<br>(1 week)	Biopsy	N/A	N/A	N/A	N/A
Joura<br>1992<br>Austria	Case 1	83	post-<br>menopausal*	Vaginal<br>bleeding	Biopsy	- CXR<br>- Cystoscopy	nil	- Recurrence (NOS)<br>- Lung<br>(5 months post initial management)	5 months disease free survival<br>15 months overall survival<br>(dead)
Pinedo<br>1991<br>Spain	Case 1	30	pre-<br>menopausal	Vaginal<br>bleeding	Biopsy	N/A	nil	nil	34 months disease free survival<br>34 months overall survival

									(alive)
Vleugels<br>1990<br>Netherlands	Case 1	64	post-menopausal	Vaginal discharge<br>(3 weeks)	Biopsy	- CT	nil	nil	48 months disease free survival<br>48 months overall survival<br>(alive)
Owens<br>1988<br>UK	Case 1	58	post-menopausal	Vaginal bleeding<br>(2 months)	Biopsy	- CXR	- Pelvic wall	- Lung, Bones<br>(3 months post-initial management)	9 months overall survival<br>(dead)
Krishnamoorhty<br>1986<br>UK	Case 1	74	post-menopausal	Vaginal bleeding	Cytology	- Biopsy<br>- IV pyelogram<br>- CT<br>- CXR<br>- Radiological assessment (NOS)	nil	N/A	N/A
Mudge<br>1981<br>UK	Case 1	52	pre-menopausal	Vaginal bleeding	Cytology	N/A	N/A	- Muscle<br>- Metastatic disease (NOS)<br>(12 months post-surgery)	12 months disease free survival<br>18 months overall survival<br>dead
Genton<br>1981<br>Switzerland	Case 1	45	N/A	Vaginal bleeding<br>(3 months)	Curettage	- CXR<br>- Urogram<br>- Cystoscopy<br>- US<br>- CT	nil	N/A	N/A
Evers<br>1950<br>UK	Case 1	62	post-menopausal*	Vaginal bleeding<br>(2 weeks)	Biopsy	- CXR	N/A	N/A	1 month overall survival<br>(dead)
Americas									

Nai 2018 Brazil	Case 1	35	pre-menopausal	Vaginal bleeding (2 months)	Biopsy	- PET-CT	- Liver - Kidney	- Liver	12 months overall survival (dead)
Schiavone 2016 USA	Case 1	62	post-menopausal*	Vaginal bleeding	N/A	N/A	nil	- Lymph nodes (6 months post initial management)	6 months disease free survival 19 months overall survival (alive)
Berger 2015 USA	Case 1	54	post-menopausal	N/A	Biopsy	- MRI - PET-CT	nil	- Liver - Lung (8 weeks post initial management)	7 months overall survival (dead)
Calderón-Salazar 2011 Mexico	Case 1	34	pre-menopausal	Vaginal bleeding (2 months)	Biopsy	- Cystoscopy - Colonoscopy - CT	nil	nil	96 months disease free survival 98 months overall survival (alive)
Setia 2010 USA	Case 1	72	post-menopausal*	N/A	N/A	N/A	N/A	N/A	N/A
	Case 2	50	N/A	Postcoital vaginal bleeding	N/A	N/A	N/A	N/A	N/A
Jin 2007 USA	Case 1	63	post-menopausal*	N/A	Cytology	- Biopsy	nil	- Recurrence (NOS) (12 months post-surgery)	12 months disease free survival
Clark 1999 USA	Case 1	63	post-menopausal*	Vaginal bleeding	Biopsy	N/A	nil	- Pelvis - Ureter (7 months post-surgery)	7 months disease free survival 14 months overall survival (dead)

Cantuaria 1999 USA	Case 1	70	post-menopausal*	Vaginal bleeding (4 months)	Punch biopsy	N/A	nil	- Adrenal glands (12 months post-radiotherapy)	15 months disease free survival 29 months overall survival (dead)
Schlosshauer 1998 USA	Case 1	88	post-menopausal	Vaginal Discharge, weight loss and haematuria (several months)	Cytology	- Radiological assessment (NOS)	nil	- Metastatic disease (NOS)	9 months overall survival (dead)
Teixeira 1998 Brazil	Case 1	65	post-menopausal*	Vaginal Bleeding and pelvic pain (2 months)	US	- Biopsy<br>- Sigmoidoscopy<br>- Cystoscopy<br>- CXR<br>- CT	nil	N/A	6 months overall survival (dead)
Kristiansen 1992 USA	Case 1	72	post-menopausal	Vaginal bleeding	Biopsy	- CT	- Lung	N/A	12 months overall survival (alive)
Santoso 1990 USA	Case 1	35	pre-menopausal	Vaginal bleeding	CT	- Sigmoidoscopy<br>- Cystoscopy<br>- Biopsy	nil	nil	17 months disease free survival 17 months overall survival (alive)
	Case 2	58	post-menopausal	Vaginal bleeding	Cytology	- Biopsy (vagina)<br>- CT<br>- MRI<br>- CXR	nil	nil	5 months disease free survival 5 months overall survival (alive)
Podczaski	Case 1	71	post-	N/A	Biopsy	- Biopsy	N/A	- Clitoris, Cervix, Urethra, Peritoneum	N/A

1990<br>USA◊			menopausal*		(labia)	- Colposcopy			
Holmquist<br>1988<br>USA	Case 1	46	pre-menopausal	Vaginal bleeding<br>(1 month)	Cytology	- Biopsy<br>- CXR<br>- CT<br>- IV pyelogram<br>- Cystoscopy<br>- Radiological assessment (NOS)	nil	N/A	N/A
Yu<br>1987<br>USA	Case 1	51	post-menopausal	Vaginal bleeding, discharge and right hemiparesis<br>(3 months)	CXR	- CT<br>- Cytology	- Basal ganglia<br>- Brain	N/A	5 months overall survival<br>(dead)
Ramsey<br>1981<br>USA	Case 1	65	post-menopausal	Vaginal bleeding<br>(1 month)	Biopsy	N/A	- Lymph nodes	- Mesentery, Liver, Lung<br>- Lymph nodes<br>(6 months post-surgery)	6 months disease free survival<br>10 months overall survival<br>(dead)
Hall<br>1980<br>USA	Case 1	26	pre-menopausal	Vaginal bleeding<br>Vaginal discharge<br>(3 weeks)	Biopsy	- CXR<br>- liver isotope scan<br>- Urogram<br>- Sigmoidoscopy<br>- Cystoscopy<br>- Lymphangiogram<br>- CT<br>- US	nil	N/A	11 months overall survival<br>(alive)

Puri 1976 USA	Case 1	70	post-menopausal	Vaginal bleeding	Biopsy	- Laboratory tests (NOS)	nil	- Mediastinal, Liver, Lung, Retroperitoneal, Mesentery, Pancreas<br>- Pelvis<br>- Lymph nodes<br>(6 weeks post-surgery)	11 months overall survival<br>(died)
Jones 1971 USA	Case 1	39	N/A	Asymptomatic	Cytology	N/A	N/A	- Vagina, Urethra, Vulva, Brain, Lung, Bowel<br>- Lymph nodes<br>(11 years post-surgery)	132 months disease free survival<br>168 months overall survival<br>(dead)
Africa									
Bennani 2013 Morocco	Case 1	40	pre-menopausal*	Vaginal bleeding (3 months)	US	- MRI<br>- Biopsy<br>- CT	- Lung	N/A	N/A
Zamiati 2001 Morocco	Case 1	35	pre-menopausal	Vaginal bleeding	Biopsy	- CXR<br>- TAUS<br>- Rectoscopy<br>- Cystoscopy	nil	- Peritoneum<br>- Breast<br>(5 months post initial management**)	10 months overall survival<br>(dead)
Oceania									
Fleming 1994 Australia	Case 1	70	post-menopausal	Vaginal bleeding	Cytology	- Biopsy<br>- Colposcopy	nil	N/A	18 months disease free survival<br>18 months overall survival<br>(alive)

<u>Abbreviations</u>

CT: Computed tomography

CXR: chest X-ray

F: Female

M: Male

MRI: Magnetic Resonance Imaging

N/A: Not available

NOS: not otherwise specified

US: ultrasound

Footnotes

◊ Primary source of MM not clearly specified

^Case 3 was 18 weeks pregnant at diagnosis

*Women older than 55 years and younger than 40 years were considered as post-menopausal and pre-menopausal, respectively

**Initial management not specified by the authors

Table S3. Staging and therapeutic management of included patients.

Author, Year, Country	Case	Age	Staging	Initial Management	Secondary Management (adjuvant)	Further Management (post recurrence)
Sone 2022 Japan	Case 1	73	FIGO IIIC1	• Radical hysterectomy<br>• Salpingo-oophorectomy<br>• Pelvic lymphadenectomy	• Radiotherapy	• Radiotherapy<br>• Immunotherapy (nivolumab, ipilimumab)
Ng 2021 China	Case 1	64	FIGO IIIA	• Total hysterectomy<br>• Salpingo-oophorectomy<br>• Pelvic lymphadenectomy	nil	nil
Suzuki 2021 Japan	Case 1	74	AJCC IIC	• Radical hysterectomy<br>• Salpingo-oophorectomy	• Immunotherapy (pembrolizumab)	• Supportive care
Cai 2021 China	Case 1	48	clinical Stage I*	N/A	N/A	N/A
	Case 2	42	clinical stage IV*	N/A	N/A	N/A
	Case 3	55	clinical stage IV*	N/A	N/A	N/A
	Case 4	42	clinical stage II*	N/A	N/A	N/A
	Case 5	34	clinical stage III*	N/A	N/A	N/A
	Case 6	54	clinical stage IV*	N/A	N/A	N/A
Diakosavvas 2020	Case 1	34	FIGO IB1	• Radical hysterectomy<br>• Salpingo-oophorectomy<br>• Pelvic lymphadenectomy	N/A	• Immunotherapy (nivolumab)

Greece						
Pumpure 2020 Latvia	Case 1	25	FIGO IVA	• Radical hysterectomy<br>• Partial omenectomy<br>• Pelvic lymphadenectomy	• Immunotherapy (rigvir)	• Node excision<br>• Hemicolectomy<br>• Lymphadenectomy
Anko 2020 Japan	Case 1	54	FIGO IB1	• Radical hysterectomy<br>• Salpingo-oophorectomy<br>• Pelvic lymphadenectomy	nil	• Immunotherapy (nivolumab)
Shakeel 2020 Pakistan	Case 1	68	T4NxM0	N/A	N/A	N/A
Indini 2019 Italy	Case 1	61	FIGO IIC	• Complete resection (NOS)	• Immunotherapy (ipilimumab)	N/A
Pang 2019 China	Case 1	58	T2N0M1**	• Radical hysterectomy<br>• Salpingo-oophorectomy<br>• Pelvic lymphadenectomy<br>• Partial urethrectomy<br>• Total vaginectomy	• Chemotherapy (DTIC, nedaplatib)<br>• Biological therapy (IFNα-2b)	nil
Yin 2019 China	Case 1	55	FIGO IIA	• Radiotherapy<br>• Chemotherapy<br>• Radical hysterectomy<br>• Salpingo-oophorectomy<br>• Pelvic lymphadenectomy	• Chemotherapy	• Chemotherapy (dimethyl triazemo imidazole, carboxamide, Nedaplatine, Navelbine)
	Case 2	81	N/A	Patient refused treatment	nil	• Nil
Eniu 2019 Romania	Case 1	51	Stage I*	• Radical hysterectomy<br>• Salpingo-oophorectomy<br>• Pelvic lymphadenectomy	nil	• Chemotherapy (DTIC)

Srivastava 2018 India	Case 1	42	N/A	• Total hysterectomy<br>• Salpingo-oophorectomy	• Chemotherapy (paclitaxel, carboplatin)	• Chemotherapy (cisplatin, vinblastine, DTIC)
Sun 2018 China	Case 1	56	FIGO IB1	• Radical hysterectomy<br>• Salpingo-oophorectomy	• Chemotherapy	N/A
	Case 2	62	FIGO IIB	• Radical hysterectomy<br>• Salpingo-oophorectomy	• Chemotherapy<br>• Radiotherapy<br>• Immunotherapy	N/A
	Case 3	38	FIGO IVB	Nil	nil	N/A
	Case 4	62	FIGO IB1	• Radical hysterectomy<br>• Salpingo-oophorectomy	nil	N/A
	Case 5	53	FIGO IB1	• Radical hysterectomy<br>• Salpingo-oophorectomy	• Argon helium knife<br>• Chemotherapy (sunitinib)	N/A
	Case 6	57	FIGO IIB	• Chemotherapy<br>• Immunotherapy	nil	N/A
	Case 7	80	FIGO IIIB	nil	nil	N/A
	Case 8	54	FIGO IB1	• Radical hysterectomy<br>• Salpingo-oophorectomy	nil	N/A
	Case 9	50	FIGO IIA2	• Total hysterectomy	nil	N/A
	Case 10	58	FIGO IIA	• Total hysterectomy	• Radiotherapy	N/A
	Case 11	45	FIGO IB1	• Radical hysterectomy<br>• Salpingo-oophorectomy<br>• Pelvic lymphadenectomy	• Chemotherapy	N/A
	Case 12	55	FIGO IIB	• Radical hysterectomy<br>• Salpingo-oophorectomy	• Chemotherapy<br>• Radiotherapy	N/A
	Case 13	60	FIGO IB1	• Radical hysterectomy<br>• Salpingo-oophorectomy	nil	N/A
	Case 14	69	FIGO IB1	• Radical hysterectomy<br>• Excision of vulva<br>• Partial urethrectomy	• Chemotherapy<br>• Immunotherapy	N/A

Kim 2018 Korea	Case 1	40	N/A	• Radical hysterectomy<br>• Salpingo-oophorectomy<br>• Pelvic lymphadenopathy<br>• Partial vaginectomy	• Immunotherapy (pembrolizumab)	• Immunotherapy (pembrolizumab)
Nai 2018 Brazil	Case 1	35	N/A	• Chemotherapy	• Immunotherapy (ipilimumab, nivolumab)	• Radiotherapy
Yuan 2017 China	Case 1	61	FIGO IIB	• Radical hysterectomy<br>• Pelvic lymphadenectomy	• Biological therapy (IL-2, IFNα)	N/A
	Case 2	74	FIGO IB2	• Radical hysterectomy<br>• Pelvic lymphadenectomy	• Chemotherapy (DTIC and cisplatin)	nil
	Case 3	56	FIGO IIIB	• Chemotherapy<br>• Radiotherapy	Nil	nil
	Case 4	74	FIGO IIA1	• Total hysterectomy<br>• Pelvic lymphadenectomy	• Chemotherapy (DTIC, cisplatin)<br>• Biological therapy (IL-2, IFNα)	• Chemotherapy
	Case 5	77	FIGO IB1	• Total hysterectomy	nil	• Surgery (NOS)<br>• Chemotherapy (DTIC, cisplatin)
	Case 6	45	FIGO IB1	• Radical hysterectomy<br>• Pelvic lymphadenectomy	nil	nil
	Case 7	50	FIGO IB2	• Total hysterectomy<br>• Pelvic lymphadenectomy	• Chemotherapy (DTIC, cisplatin)	• Surgery (NOS)<br>• Chemotherapy
	Case 8	58	FIGO IIB1	• Radical hysterectomy<br>• Pelvic lymphadenectomy	• Chemotherapy (DTIC, cisplatin)	• Surgery (NOS)<br>• Chemotherapy
	Case 9	57	FIGO IIA1	• Chemotherapy	nil	N/A
	Case 10	42	FIGO IIB	• Radiotherapy (preoperative)<br>• Total hysterectomy	• Radiotherapy	nil

	Case 11	63	FIGO IIIB	• Chemotherapy<br>• Radiotherapy	nil	N/A
	Case 12	54	FIGO IIB	• Radiotherapy (preoperative)<br>• Chemotherapy<br>• Radical hysterectomy<br>• Pelvic lymphadenectomy	• Chemotherapy (DTIC, cisplatin)<br>• Radiotherapy	nil
	Case 13	78	FIGO IIIB	• Chemotherapy<br>• Radiotherapy	nil	N/A
	Case 14	68	FIGO IIA1	• Radical hysterectomy<br>• Pelvic lymphadenectomy	nil	• Surgery (NOS)
Julião 2017 Portugal	Case 1	64	FIGO IIB	• Radical hysterectomy<br>• Pelvic lymphadenectomy	• Brachytherapy	N/A
Noguchi 2017 Japan	Case 1	66	FIGO IIIA	• Radical hysterectomy<br>• Salpingo-oophorectomy<br>• Pelvic lypmphadectomy<br>• Total vaginectomy	• Chemotherapy	• Immunotherapy (nivolumab)
Lim 2017 Singapore	Case 1	47	FIGO IB2	• Radical hysterectomy<br>• Salpingectomy<br>• Pelvic lymphadenectomy<br>• Transposition of ovaries	• Chemotherapy<br>• Radiotherapy	N/A
Gupta 2016 India	Case 1	68	FIGO IB2	• Surgery (NOS)	nil	nil
Lee 2016 Korea	Case 1	70	N/A	• Focal argon laser	nil	nil
Arık 2016 Turkey	Case 1	61	FIGO IB1	• Radical hysterectomy<br>• Salpingo-oopherectomy<br>• Retroperitoneal and pelvic lymphadenectomy	• Radiotherapy<br>• Chemotherapy (cisplatin)	N/A

Ferraioli 2016 France	Case 1	74	FIGO IIB	• Radiotherapy<br>• Surgery (NOS)<br>• Retroperitoneal and pelvic lymphadenectomy	nil	• Chemotherapy
Schiavone 2016 USA	Case 1	62	Ballantyne Stage I	• Immotherapy (ipilimumab)<br>• Radiotherapy<br>• Hysterectomy<br>• Salpingo-oophorectomy<br>• Partial vaginectomy	nil	• Immunotherapy (pembrolizumab)
Berger 2015 USA	Case 1	54	N/A	• Radical hysterectomy<br>• Salpingo-oophorectomy<br>• Pelvic lymphadenectomy	• Brachytherapy<br>• Immunotherapy (ipilimumab)	nil
Geredeli 2015 Turkey	Case 1	73	N/A	• Radical hysterectomy<br>• Salpingo-oophorectomy<br>• Pelvic and para-aortic lymphadenectomy<br>• Partial omentectomy	• Biological therapy (IFN α2b)	• Chemotherapy
Cetinkaya 2015 Turkey	Case 1	43	FIGO IB1	• Radical hysterectomy<br>• Pelvic and para-aortic lymphadenectomy	• Radiotherapy<br>• Biological therapy (interferon)	N/A
Mihmanli 2015 Turkey	Case 1	66	N/A	• Radical hysterectomy<br>• Salpingo-oophorectomy<br>• Pelvic lymphadenectomy		N/A
Bhargava 2014 India	Case 1	35	N/A	N/A	N/A	N/A

Liu<br>2014<br>China	Case 1	65	FIGO IB1	• Chemotherapy (cisplatin, vincristine, DTIC)<br>• Radical hysterectomy<br>• Salpingo-oophorectomy<br>• Pelvic lymphadenectomy	• Chemotherapy	nil
Min<br>2014<br>South Korea	Case 1	46	FIGO IB2	• Chemotherapy (cisplatin, DTIC)<br>• Radical hysterectomy<br>• Right salpingo-oophorectomy<br>• Pelvic lymphadenectomy	• Chemotherapy (cisplatin, DTIC)<br>• Radiotherapy	nil
Omranipour<br>2014<br>Iran	Case 1	49	FIGO IIA2	• Total hysterectomy<br>• Salpingo-oophorectomy<br>• Pelvic lymphadenectomy	N/A	N/A
Shenjere<br>2014<br>UK	Case 1	51	N/A	• Chemotherapy (ifosfamide, vincristine, actinomycin, doxorubicin)<br>• Total hysterectomy<br>• Salpingo-oophorectomy<br>• Pelvic lymphadenectomy	• Chemotherapy (ifosfamide, vincristine, actinomycin)	N/A
Shrivastava<br>2014<br>India	Case 1	42	FIGO IVA	• Radiation	nil	N/A
Myriokefalitaki<br>2013<br>UK	Case 1	63	FIGO IIA1	• Radical hysterectomy<br>• Salpingo-oophorectomy<br>• Pelvic lymphadenectomy<br>• Partial vaginectomy	nil	nil

Singh<br>2013<br>India	Case 1	35	FIGO IIA	• Radical hysterectomy (attempted/not feasible)<br>• Pelvic and para-aortic lymphadenectomy	• Chemotherapy (cisplatin, DTIC, vinblastine)	N/A
Bennani<br>2013<br>Morocco	Case 1	40	N/A	• Chemotherapy (DTIC)	N/A	N/A
Parada<br>2012<br>Spain	Case 1	76	N/A	• Total hysterectomy<br>• Salpingo-oophorectomy<br>• Iliac and paraaortic lymphadenectomy	N/A	N/A
Tsai<br>2012<br>Taipei	Case 1	66	N/A	• Radical hysterectomy<br>• Total vaginectomy<br>• Pelvic lymphadenectomy	nil	• Surgery (NOS)<br>• Radiotherapy<br>• Chemotherapy
Calderón-Salazar<br>2011<br>Mexico	Case 1	34	N/A	• Total pelvic exenteration<br>• Colostomy<br>• Bricker ileal conduit	• Radiotherapy	nil
Zhang<br>2011,<br>China	Case 1	67	FIGO IB1	• Radical hysterectomy<br>• Salpingo-oophorectomy<br>• Pelvic lymphadenectomy<br>• Partial vaginectomy	nil	N/A
Simões<br>2011<br>Portugal	Case 1	75	FIGO IB1	• Radical hysterectomy<br>• Salpingo-oophorectomy<br>• Pelvic lymphadenectomy	nil	• Chemotherapy<br>• Radiotherapy
Das<br>2010<br>India	Case 1	40	N/A	• Hysterectomy	N/A	N/A
	Case 2	61	N/A	• Hysterectomy	N/A	N/A

Setia 2010 USA	Case 1	72	N/A	• Surgery (NOS)	• Radiotherapy	N/A
	Case 2	50	N/A	• Surgery (NOS)	• Radiotherapy	N/A
Duggal 2010, India	Case 1	65	Stage I*	nil	nil	nil
Khurana 2009 India	Case 1	58	N/A	• Chemotherapy (cisplatin)<br>• Radical hysterectomy<br>• Pelvic lymphadenectomy	nil	nil
An 2009 China	Case 1	67	FIGO IIA	• Total hysterectomy<br>• Salpingo-oophorectomy<br>• Pelvic lymphadenectomy<br>• Partial vaginectomy	• Chemotherapy (temozolomide)<br>• Biological therapy (IL-2, INFα)	nil
Baruah 2009 India	Case 1	40	FIGO IIa	• Radical hysterectomy<br>• Pelvic lymphadenectomy	• Chemotherapy (Cisplatin and DTIC)	• Radiotherapy
Yücesoy 2009 Turkey	Case 1	61	FIGO IB1	• Radical hysterectomy<br>• Salpingo-oopherectomy<br>• Retroperitoneal and pelvic lymphadenectomy	nil	nil
Mandato 2009 Slovenia	Case 1	61	FIGO IB1	• Radical hysterectomy<br>• Salpingo-oophorectomy<br>• Pelvic lymphadenectomy	nil	nil
Pusceddu 2008 Italy	Case 1	43	FIGO IIB	• Chemotherapy (epirubicin, ifosfamide)<br>• Total hysterectomy<br>• Salpingo-oophorectomy	nil	• colpectomy, pelvic and paraaortic lymphadenectomy<br>• Chemotherapy (cisplatin, vindesine, DTIC, fotemustine)
Jin 2007	Case 1	63	N/A	• Trachelectomy<br>• Pelvic lymphadenectomy	nil	N/A

USA						
Mousavi 2006 Iran	Case 1	38	FIGO IB1	• Radical hysterectomy<br>• Salpingo-oophorectomy<br>• Retroperitoneal and pelvic lymphadenectomy	• Radiotherapy	nil
Wydra 2006 Poland	Case 1	54	FIGO IIA	• Radical hysterectomy	• Radiotherapy<br>• Chemotherapy (DTIC)	• Chemotherapy (DTIC)
Ma 2005 China	Case 1	45 (mean)	N/A	• Chemotherapy (cisplatin, epirubicin, ifosfamide, taxol, carboplatin)<br>• Radiotherapy<br>• Radical hysterectomy<br>• Pelvic lymphadenectomy	nil	N/A
	Case 2		N/A	• Radical hysterectomy<br>• Pelvic lymphadenectomy	• Chemotherapy (cisplatin, cyclophosphamide, etoposide)	N/A
	Case 3		N/A	• Radical hysterectomy<br>• Pelvic lymphadenectomy	nil	N/A
	Case 4		N/A	• Radical hysterectomy<br>• Pelvic lymphadenectomy	• Chemotherapy (cisplatin, DTIC, carmustin)<br>• Biological therapy (IFN-γ, IL-2)	N/A
Gupta 2005 India	Case 1	39	N/A	• Radical hysterectomy<br>• Pelvic lymphadenectomy	• Radiotherapy	• Chemotherapy (dacarbazepine, cisplatin)
Siozos 2005	Case 1	82	N/A	• Radiotherapy	nil	N/A

UK						
Kudrimoti<br>2004<br>India	Case 1	45	N/A	• Cervical stump excision	N/A	N/A
Makovitzk<br>y<br>2003<br>Germany	Case 1	67	N/A	• Radical hysterectomy<br>• Salpingo-oophorectomy<br>• Inguinal and Pelvic lymphadenectomy	• Radiotherapy<br>• Biological therapy (IFN-a2b)	nil
Boldt<br>2003<br>Germany	Case 1	26	FIGO IIIA	• Hysterectomy<br>• Salpingo-oophorectomy	• Biological therapy (IFN)	N/A
	Case 2	70	FIGO IB	• Cervical stump excision<br>• Vaginal cuff<br>• Pelvic lymphadenectomy	N/A	N/A
Gupta<br>2003<br>India	Case 1	70	N/A	• Chemotherapy	nil	nil
Deshpande<br>2001<br>India	Case 1	50	FIGO I	• Radical hysterectomy<br>• Pelvic lymphadenectomy	• Radiotherapy	nil
Zamiati<br>2001<br>Morocco	Case 1	35	FIGO IIA	• Radical hysterectomy<br>• Salpingo-oophorectomy<br>• Pelvic lymphadenectomy	nil	N/A
Furuya<br>2001<br>Japan	Case 1	33	FIGO IIB	• Radical hysterectomy<br>• Salpingo-oophorectomy<br>• Retroperitoneal lymphadenectomy	nil	nil
Benson<br>2000<br>UK	Case 1	73	N/A	• Radiotherapy	nil	nil
Wasef<br>1999	Case 1	71	N/A	• Radical hysterectomy<br>• Pelvic lymphadenectomy	nil	• Chemotherapy (bleomycin)

UK						
Clark<br>1999<br>USA	Case 1	63	N/A	• Total hysterectomy<br>• Salpingo-oophorectomy	nil	• Chemotherapy (DTIC, cisplatin)
Cantuaria<br>1999<br>USA	Case 1	70	N/A	• Radical hysterectomy<br>• Salpingo-oophorectomy<br>• Pelvic lymphadenectomy<br>• Partial vaginectomy	• Radiotherapy	• Biological therapy (IFN)
Takehara<br>1999<br>Japan	Case 1	76	FIGO Ib	• Radiotherapy<br>• Radical hysterectomy<br>• Salpingo-oophorectomy<br>• Pelvic lymphadenectomy	nil	nil
Schlosshau er<br>1998<br>USA	Case 1	88	N/A	• Radiotherapy	N/A	N/A
Teixeira<br>1998<br>Brazil	Case 1	65	FIGO IIIB	• Radiotherapy	nil	N/A
Ishikura<br>1998<br>Japan	Case 1	51	N/A	• Surgery (NOS)	• Biological therapy (IFN-α)<br>• BCG<br>• Chemotherapy (cyclophosphamide, adriamycin, cisplatin)	N/A
Miyagi<br>1997<br>Japan	Case 1	57	N/A	• Laparotomy<br>• Hysterectomy<br>• Pelvic lymphadenectomy	nil	• Chemotherapy (DTIC, ACNU, VCR, cisplatin)
Butt<br>1996<br>UK	Case 1	72	N/A	• Cervical amputation<br>• Local excision of vulval lesions	N/A	N/A

Fleming 1994 Australia	Case 1	70	N/A	• Radical hysterectomy	nil	N/A
Moon 1993 Korea	Case 1	65	N/A	• Total hysterectomy<br>• Salpingo-oophorectomy	• Radiotherapy	N/A
Joura 1992 Austria	Case 1	83	FIGO IIIAB	• Radiotherapy	N/A	• Radiotherapy
Kristiansen 1992 USA	Case 1	72	FIGO IV	• Radiotherapy<br>• Hysterectomy<br>• Salpingo-oophorectomy<br>• Partial vaginectomy	nil	nil
Khoo 1991 Hong Kong	Case 1	62	FIGO III	• Radiotherapy	• Chemotherapy (DTIC)	N/A
	Case 2	60	FIGO III	• Radiotherapy<br>• Radical hysterectomy	• Chemotherapy (DTIC)	N/A
	Case 3	37	FIGO IB	• Caesarean section and radical hysterectomy	N/A	N/A
Pinedo 1991 Spain	Case 1	30	FIGO IB	• Radical hysterectomy<br>• Salpingo-oophorectomy<br>• Pelvic lymphadenectomy<br>• Partial vaginectomy	• Radiotherapy	nil
Santoso 1990 USA	Case 1	35	N/A	• Radical hysterectomy<br>• Pelvic lymphadenectomy<br>• Transposition of ovaries	nil	nil
	Case 2	58	N/A	• Radical hysterectomy<br>• Salpingo-oophorectomy<br>• Pelvic lymphadenectomy	nil	nil
Vleugels 1990 Netherlands	Case 1	64	N/A	• Radical hysterectomy<br>• Salpingo-oophorectomy<br>• Pelvic lymphadenectomy	nil	nil

Podczaski 1990 USA	Case 1	71	N/A	• Radical hemi vulvectomy<br>• Pelvic node dissection<br>• Inguinal and femoral lymphadenectomy	nil	• Vaginal hysterectomy
Chua 1989 Singapore	Case 1	52	N/A	• Radical hysterectomy<br>• Partial vaginectomy	nil	• Vulvectomy<br>• Vaginectomy<br>• Radiotherapy
Holmquist 1988 USA	Case 1	46	FIGO IIIA	• Radiotherapy<br>• Chemotherapy	nil	N/A
Owens 1988 UK	Case 1	58	FIGO IIIB	• Radiotherapy	• Serial biopsies during course of radiotherapy	• Radiotherapy
Yu 1987 USA	Case 1	51	N/A	• Endometrial curettage and excision of endocervical mass	• Radiotherapy	N/A
Krishnamo orhty 1986 UK	Case 1	74	N/A	• Radical hysterectomy<br>• Pelvic lymphadenectomy	nil	N/A
Ramsey 1981 USA	Case 1	65	N/A	• Radical hysterectomy<br>• Pelvic lymphadenectomy	• Chemotherapy (phenylalanine mustard)	• Surgery (NOS)
Mudge 1981 UK	Case 1	52	N/A	• Cervical mass excision<br>• Endometrial curettage	• Total hysterectomy<br>• Salpingo-oophorectomy	N/A
Genton 1981	Case 1	45	N/A	• Cervical mass excision	• Radical hysterectomy<br>• Pelvic lymphadenectomy	N/A

Switzerland					• Partial vaginectomy	
Hall 1980 USA	Case 1	26	FIGO IIB	• Pelvic exenteration	nil	N/A
Puri 1976 USA	Case 1	70	FIGO IIB	• Radiotherapy<br>• Total hysterectomy<br>• Salpingo-oophorectomy	nil	• Chemotherapy (vinblastin, bleomycin)
Jones 1971 USA	Case 1	39	N/A	• Radical hysterectomy<br>• Salpingo-oophorectomy<br>• Pelvic lymphadenectomy<br>• Partial vaginectomy	nil	• Vulvectomy<br>• Chemotherapy (meplhalan, imidazole, carboxamide, 5-fluorouracil)
Evers 1950 UK	Case 1	62	N/A	• Radiotherapy<br>• Radical hysterectomy	N/A	N/A

Abbreviations
CT: Computed tomography
CXR: chest X-ray
M: Male
N/A: Not available
NOS: not otherwise specified
MRI: Magnetic Resonance Imaging
ix: investigations, B/L: bilateral, RT: radiotherapy, DTIC: dacarbazine, ACNU:, VCR: Vincristine
RLL: Right lower limb

Footnotes
*staging method not specified by the author
**Primary source of MM to clearly specified

Table S4. Quality assessment of the included studies.

Author, year	Q1	Q2	Q3	Q4	Q5	Q6	Q7	Q8
Sone, 2022	●	●	⦰	●	●	●	●	●
Cai, 2021	⦰	○	●	●	●	○	○	●
Ng, 2021	⦰	⦰	○	⦰	⦰	●	◌	●
Suzuki, 2021	●	●	⦰	●	●	●	●	●
Anko, 2020	⦰	⦰	●	●	●	⦰	●	●
Diakosavvas, 2020	●	●	●	●	●	●	◌	●
Pumpure, 2020	●	●	●	●	●	●	●	●
Eniu, 2019	●	●	●	○	●	●	●	●
Indini, 2019	⦰	●	○	○	●	●	◌	●
Shakeel, 2020	⦰	●	⦰	●	●	●	◌	●
Pang, 2019	⦰	●	⦰	●	●	●	◌	●
Yin, 2019	○	○	○	⦰	●	●	◌	●
Kim, 2018	⦰	○	○	●	●	●	●	●
Nai, 2018	●	●	○	●	●	●	◌	●
Srivastava, 2018	○	○	●	●	●	●	●	●
Sun 2018	⦰	○	○	○	●	●	○	●
Julião, 2017	⦰	●	●	●	●	●	◌	●
Lim, 2017	⦰	○	●	⦰	●	●	◌	●
Noguchi, 2017	○	○	●	⦰	●	●	●	●

Yuan, 2017	○	○	●	○	●	●	○	●
Arik, 2016	●	●	●	●	●	●	●	●
Ferraioli, 2016	○	○	●	○	●	●	○	●
Gupta, 2016	⦰	○	⦰	●	⦰	●	◌	●
Lee, 2016	⦰	○	○	●	●	●	●	●
Schiavone, 2016	●	○	●	○	●	●	●	●
Berger, 2015	⦰	○	●	○	●	●	●	●
Cetinkaya, 2015	○	●	⦰	○	●	●	◌	●
Geredeli, 2015	●	○	○	●	●	●	●	●
Mihmanli, 2015	⦰	○	○	●	●	⦰	◌	●
Bhargava, 2014	●	○	○	●	○	○	○	●
Liu, 2014	○	○	○	●	●	●	◌	●
Min, 2014	⦰	●	●	●	●	●	◌	●
Omranipour, 2014	⦰	●	⦰	●	●	○	◌	●
Shenjere, 2014	⦰	●	●	●	●	○	●	●
Shrivastava, 2014	●	○	⦰	●	●	●	◌	●
Bennani, 2013	⦰	○	○	●	●	⦰	◌	●
Myriokefalitaki, 2013	●	●	●	●	●	●	◌	●
Singh, 2013	●	○	●	●	●	●	◌	●
Parada, 2012	●	●	●	●	●	⦰	⦰	●
Tsai, 2012	⦰	○	○	●	●	⦰	●	●

Calderón-Salazar, 2011	●	○	○	●	●	●	●	●
Simões, 2011	⊘	○	⊘	●	●	●	●	●
Zhang, 2011	⊘	○	⊘	●	●	○	○	●
Das,2010	⊘	○	●	○	○	○	○	●
Duggal, 2010	⊘	●	●	●	●	○	○	●
Setia, 2010	●	○	⊘	⊘	●	○	○	●
An, 2009	⊘	○	⊘	⊘	●	●	●	●
Baruah, 2009	⊘	●	⊘	●	●	●	●	●
Khurana, 2009	⊘	○	○	●	●	●	●	●
Mandato, 2009	⊘	●	○	○	●	●	○	●
Yücesoy, 2009	⊘	●	●	●	●	●	○	●
Pusceddu, 2008	●	○	●	⊘	●	●	●	●
Jin, 2007	●	●	●	●	●	●	○	●
Mousavi, 2006	⊘	⊘	⊘	●	●	●	○	●
Wydra, 2006	⊘	○	●	●	●	●	●	●
Gupta, 2005	⊘	○	○	●	●	●	●	●
Ma, 2005	○	○	○	⊘	●	●	○	●
Siozos, 2005	⊘	●	⊘	●	●	○	○	●
Kudrimoti, 2004	⊘	○	⊘	●	○	○	○	●
Gupta, 2003	⊘	●	●	●	⊘	●	○	●
Boldt, 2003	⊘	●	○	●	●	●	○	●

Makovitzky, 2003	⊘	○	○	●	●	●	◌	●
Deshpande, 2001	⊘	○	●	●	●	●	◌	●
Furuya, 2001	●	○	●	●	●	●	◌	●
Zamiati, 2001	⊘	●	●	●	●	●	●	●
Benson, 2000	⊘	●	⊘	●	●	●	●	●
Cantuaria, 1999	●	●	●	○	●	●	●	●
Clark, 1999	●	○	●	⊘	●	●	●	●
Takehara, 1999	●	●	●	●	●	●	◌	●
Wasef, 1999	⊘	●	●	●	●	●	●	●
Ishikura, 1998	●	○	○	○	●	●	◌	●
Schlosshauer, 1998	●	○	○	●	⊘	●	●	●
Teixeira, 1998	⊘	○	●	●	●	●	◌	●
Miyagi, 1997	●	●	●	●	●	●	●	●
Butt, 1996	●	●	⊘	●	○	○	◌	●
Fleming, 1994	⊘	○	○	●	●	●	◌	●
Moon, 1993	⊘	●	○	●	●	○	◌	●
Joura, 1992	⊘	●	⊘	●	●	●	●	●
Kristiansen, 1992	●	●	●	●	●	●	●	●
Khoo, 1991	⊘	○	○	○	●	●	◌	●
Pinedo, 1991	●	○	●	○	●	●	◌	●
Santoso, 1990	⊘	○	○	●	●	●	◌	●

Vleugels, 1990	⦰	○	●	●	●	●	◌	●
Podczaski, 1990	●	○	⦰	●	●	○	●	●
Chua, 1989	●	●	○	●	●	●	●	●
Holmquist, 1988	⦰	○	⦰	●	○	⦰	◌	●
Owens, 1988	⦰	●	⦰	●	●	●	●	●
Yu, 1987	⦰	●	⦰	●	⦰	●	◌	●
Krishnamoorhty, 1986	●	●	●	●	●	●	◌	●
Genton, 1981	⦰	●	●	●	●	⦰	◌	●
Mudge, 1981	⦰	●	○	●	●	●	●	●
Ramsey, 1981	●	○	●	○	●	●	●	●
Hall, 1980	⦰	○	○	●	●	●	◌	●
Puri, 1976	●	●	○	○	●	●	●	●
Jones, 1971	⦰	○	○	⦰	●	●	●	●
Evers, 1950	⦰	●	●	●	●	○	◌	●

Q1: Were patient's demographic characteristics clearly described?; Q2: Was the patient's history clearly described and presented as a timeline? Q3: Was the current clinical condition of the patient on presentation clearly described?; Q4: Were diagnostic tests or methods and the results clearly described?; Q5: Was the intervention(s) or treatment procedure(s) clearly described?; Q6: Was the post-intervention clinical condition clearly described?; Q7: Were adverse events (harms) or unanticipated events identified and described?; Q8: Does the case report provide takeaway lessons? ● = Yes; ○ = No; ⦰ = Unclear; ◌ = Not Available
